# Supplementary material for: Fire suppression makes wildfires more severe and accentuates impacts of climate change and fuel accumulation
Source: Nat Commun. 2024 Mar 25;15:2412. doi: 10.1038/s41467-024-46702-0 (PMC10963776; doi:10.1038/s41467-024-46702-0)
Supplement: Supplementary file 1 — Supplementary Information [file 41467_2024_46702_MOESM1_ESM.pdf]

Supplementary Information for:
Fire suppression makes wildfires more severe and accentuates impacts of climate change and fuel accumulation

Mark R. Kreider*, Philip E. Higuera, Sean A. Parks, William L. Rice, Nadia White, Andrew J. Larson

*Corresponding author
Email: mark.kreider@umontana.edu

This PDF file includes:
Supplementary Methods
Supplementary Figures S1 to S13
Supplementary Tables S1 to S2
References for Supplementary Information

Supplementary Methods

Simulating modeling inputs

Daily fire weather

For each ignition, we simulated daily fire weather for the entire 150-day fire season—windspeed and temporally autocorrelated live and dead fuel moistures—to be subsequently used in fire spread models (Fig. S6).

Wind speed timeseries

We modeled daily (*d*) windspeeds for a given fire j (U_{j_d}; km / hr) as arising from a Weibull distribution:

$$U_{j_d} \sim \text{Weibull}(shape = 2;\ scale = 20)$$

We assumed that the wind always blew in the same direction and held the wind-adjustment factor constant at 0.15 (i.e., the ratio of mid-flame wind speed to 20-ft open wind speed; an input variable to the fire spread model).

Surface fuel moisture timeseries

In a manner conceptually similar to the methods of Finney and colleagues (1), we simulated daily 100-hr fuel moisture values for a given fire j ($FM100_{j_d}$) using a seasonal trend (δ_j) and a set of daily temporally auto-correlated random variation that fluctuates around zero (γ_j).

$$FM100_{j_d} = \exp\left(\delta_{j_d} + \gamma_{j_d}\right)$$

We modeled the seasonal trend (δ) with a general sinusoidal curve function (ε) that reaches its lowest point at the mid-point of the summer, modified by fire-specific amplitude ($\sigma_{seasonal_j}$) and mean (μ_j) values. The daily index d can take on values from 1 to 150.

$$\delta_{j_d} = \varepsilon(d) \cdot \sigma_{seasonal_j} + \mu_j$$

$$\varepsilon = -\sin\left(\frac{2\pi d}{150} - \frac{\pi}{2}\right)$$

$$\sigma_{seasonal_j} \sim \text{uniform}(min = 0.125;\ max = 0.375)$$

Because the general sinusoidal function ε has a mean of 0 when evaluated across all 150 days of the fire season, μ_j determines the mean expected value of fire season fuel moisture. To assess the effect of climate change on the effects of fire suppression via fuel aridity, we ran simulations across a span of mean values of fuel aridity (μ_j), ranging from 1.045 to 2.61. Thus, when exponentiated, the set of μ_j values corresponds to expected mean 100-hour fuel moistures ranging from 4.2% to 20.1%, corresponding to vapor pressure deficits (VPD) of 0.33 to 2.34 kPa. This is a range equivalent to 240 years of modeled VPD increase under the RCP 8.5 climate change scenario (2) (see section below detailing estimation of VPD from 100-hour fuel moisture).

To incorporate daily variation in fuel moisture (γ_{j_d}), we used an autoregressive model where values are centered around zero, autocorrelated with the previous day's fuel moisture to a certain extent (ϕ_j), and fluctuate by some level of variability (σ_{daily}). Each fire has a specific set of parameters ϕ_j and σ_{daily_j} that comes from common distributions respectively:

$$\gamma_{j_d} \sim AR(\phi_j, \sigma_{daily_j})$$

$$\phi_j \sim \text{uniform}(min = 0.8;\ max = 0.95)$$

$$\sigma_{daily_j} \sim \text{uniform}(min = 0.125;\ max = 0.150)$$

We estimated daily average 10-hour ($FM10_{j_d}$) and 1-hour ($FM1_{j_d}$) fuel moisture by subtracting 1% and 2% respectively from the 100-hour fuel moisture (3), and defined litter fuel moisture ($FMLit_{j_d}$) as equal to 1-hour fuel moisture. We set the minimum threshold of all fuel moisture values to be 1%.

$$FM10_{j_d} = FM100_{j_d} - 1$$

$$FM1_{j_d} = FMLit_{j_d} = FM100_{j_d} - 2$$

To simulate live woody and herbaceous fuel moisture, we used a sinusoidal seasonal trend modified by the scaled inverse of the daily variation in dead fuel moisture calculated above (γ_{j_d}). In addition to the following equations, we forced values of woody and herbaceous fuel moisture that fell below 60 and 30 percentage points to 60 and 30 respectively.

$$FMWoody_{j_d} = 150\left(\sin\left(\frac{2\pi d}{150} - \frac{\pi}{2}\right) + \frac{1}{2}\right) + 60 - 30 \cdot \gamma_{j_d}$$

$$FMHerb_{j_d} = 200\left(\sin\left(\frac{2\pi d}{150} - \frac{\pi}{2}\right) + \frac{1}{2}\right) + 30 - 30 \cdot \gamma_{j_d}$$

Estimated vapor pressure deficit from 100-hour fuel moisture

To aid in interpretation, we also converted 100-hour fuel moisture values to expected associated vapor pressure deficit (VPD), using a statistical relationship parameterized with empirical data. We randomly located 500 locations within the temperate conifer forest Major Habitat Type (4) in the Western contiguous U.S. At each location, we used Google Earth Engine (5) to extract daily gridMET climate data at ~4x4 km resolution (6) from May–September for the years 2010–2020 (i.e., 500 locations x 11 years x 153 days; or 841,500 daily pairs of VPD and 100-hour fuel moisture data). Using these data, we fit an exponential model to predict VPD from 100-hour fuel moisture data (Fig. S8; $R^2 = 0.56$), and used it to transform simulated 100-hr fuel moisture values to VPD (Fig. S7):

$$VPD = 4.188 - 1.285\log(FM100)$$

Canopy fuel moisture timeseries

Canopy fuel moisture, or foliar moisture content, is an important variable determining crown fire initiation and spread, and varies seasonally within a typical range of 120% and 80% in conifers (7). For each fire, we modeled variation in daily foliar moisture content (FMC_{j_d}) with the same general sinusoidal curve function (ε) as with surface fuels, scaling it to fluctuate between 120% and 80% (Fig. S8):

$$FMC_{j_d} = 20 \cdot \varepsilon + 100$$

Fuel loading

For a given ignition, we assumed continuous and uniform fuel loading, meaning that simulated fires never experienced fuel breaks and would continue burning as long as they were not suppressed (described below) and weather conditions allowed for fire spread. To test the effects of different levels of fuel accumulation on the effects of fire suppression, we ran simulations across a range of surface and canopy fuel loading levels. To create new levels of surface fuel loading, we used Fuel Model 10 (Timber—litter and understory) (8) as a base fuel model, and then scaled dead fuel, live fuel, and fuel bed depth up or down to create a new set of fuel load parameters, while preserving the relative ratios between litter, 1-, 10-, and 100-hour fuel, live herbaceous and woody fuels, and fuel bed depth (Table S1). We kept all other surface fuel variables (e.g., surface-area-to-volume ratios or moisture of extinction of dead fuels) at the original values of Fuel Model 10 (8). We also created a range of increasing canopy fuel loading and canopy bulk density values, while holding canopy base height at 2 m. Table S1 shows the 25 levels of fuel loading that we used in simulations.

Simulating fire behavior

Ignitions

For each fire, we randomly assigned an ignition day (1–150). Ignitions could smolder for up to three days, during which time fire spread would occur if the daily fuel moisture was below the moisture of extinction (25%). If fuel moisture never fell below 25% during this three-day period, the ignition did not spread and was assumed to be extinguished naturally.

Fire spread

For ignitions that spread, fire spread was assumed to continue through the final day of the fire season or until the daily fuel moisture value exceeded the moisture of extinction (25%).

We modeled daily heading (i.e., heading direction; *r* in Fig. S9) surface fire spread rate ($r_{surface_H}$) and potential crown fire spread rate (r_{crown_H}) using Rothermel's (9) surface fire spread as modified by Albini (10) and crown fire spread models presented by Finney (11). We calculated surface and crown fire spread rates in the heading direction ($r_{surface}$ and r_{crown}) using the *rothermel* function in the R package *firebehavioR* (12). We assumed a terrain slope of 40% (20.8°), and that fires never encountered one another.

We used elliptical fire shapes (7) and modeled daily fire growth based on Huygens' principle (13): that the growth of each point on the fire perimeter can be independently modeled as an expanding ellipse. The shape of this ellipse becomes longer and narrower at higher wind speeds:

we modeled the elliptical ratio (ratio of ellipse length to width; Z) using the relationship of Simard and Young (14) as reported by Finney and colleagues (7), where U is the daily 10-m open wind speed (km / h):

$$Z = e^{0.0162 \cdot U^{1.2}}$$

Following Finney and colleagues, we truncated elliptical ratios to be no more than eight (11).

We modeled two-dimensional fire spread in polar coordinate space, by converting $r_{surface_H}$ and r_{crown_H} to expansion factors at any angle θ from 0 to π on the ellipse ($r_{surface_\theta}$ and r_{crown_θ}) using equations defining elliptical shapes from Andrews (15). To scale spread rate between $r_{surface}$ and r_{crown} for windspeeds when crown fire is predicted (i.e., when $r_{surface}$ exceeds the threshold for active crown initiation, r'_{active} (16)), we used a transition function (i.e., an estimation of "crown fraction burned" or CFB) defined by Finney (11). To model CFB at any angles (CFB_θ), we converted the open windspeed U to a relative windspeed at any angle (U_θ):

$$U_\theta = \begin{cases} \cos(\theta) \cdot U & \text{if } \cos(\theta) > 0 \\ 0 & \text{if } \cos(\theta) \leq 0 \end{cases}$$

Following Finney (11), we calculated the final expansion factor (r_{final_θ}):

$$r_{final_\theta} = r_{surface_\theta} + CFB_\theta \cdot (r_{crown_\theta} - r_{surface_\theta})$$

We converted the daily expansion factor r_{final_θ} (m min^{-1}) to daily distance burned ($D_\theta{}'$; m day^{-1}) with the assumption that the fire would actively spread for half of the day length (L_d; hours):

$$D_\theta{}' = r_{final_\theta} \cdot \frac{60\ minutes}{1\ hour} \cdot \frac{L_d}{day} \cdot \frac{1}{2}$$

Where day length (L_d; hours) was estimated as (Fig. S10):

$$L_d = 2 \cdot \sin\left(\frac{d \cdot \pi}{180} + \frac{pi}{5}\right) + 8.5$$

Fire intensity & severity

Using equations from the *rothermel* function in the R package *firebehavioR* (12), we calculated two metrics of fire intensity in the direction of spread (θ) from the ignition point (15)—fireline intensity (I_θ) and flame length (L_θ). We converted heading fireline intensity to elliptical fireline intensity using equations detailed by Catchpole and colleagues (17).

We estimated fire severity by linking flame length to tree mortality and finally to Composite Burn Index (CBI), a ground-based measure of fire severity (18). Ryan and Noste (19) present a review of estimated tree mortality across size classes of two widespread tree species in the western US—ponderosa pine (*Pinus ponderosa*) and Douglas-fir (*Pseudotsuga menziesii*)—as a function of flame length. The size classes given by Ryan and Noste (19) correspond almost directly to the strata used to estimate CBI (18). We thus used mortality values of each size class to estimate average CBI for each flame length class, assuming trees of all size classes were

present in all burned areas. Estimated CBI strata severity (18) and overall CBI values are given in Table S2.

We used estimated CBI values for each flame length range to create a continuous equation relating flame length (L_θ, in meters) to CBI, with added random variability:

$$CBI_\theta = 3\left(\frac{L_\theta}{4}\right)^{0.3} + \varepsilon$$

$$\varepsilon \sim \text{normal}(0, 0.25)$$

Where CBI_θ values below zero are forced to 0, and values above three forced to 3.0 (Fig. S11). Following the precedent of others (e.g., 17), we defined "high-severity" fire as CBI values ≥ 2.25.

Simulating suppression

For the same ignition, we tested several different suppression scenarios: three regressive suppression scenarios (Maximum, Moderate, High), one progressive suppression scenario (Progressive), and one control scenario (No suppression). We simulated suppression of fires with a two-step process: 1) initial attack and 2) subsequent suppression of fires that escape initial attack (21, 22).

To simulate initial attack success, we used a modeled relationship from Hirsch and colleagues (85) that estimates probability of escape (P_{escape}) as a function of heading fireline intensity (I; kW/m) and fire size (A; ha) at the time of initial engagement.

$$P_{escape} = 4.7271 - 0.9325 \cdot A - 0.00051 \cdot I - 0.000017 \cdot A \cdot I$$

For regressive suppression scenarios, we used different times of initial engagement, and thus fire size (Moderate = 4 hours; High = 2 hours; Maximum = 1 hour). We then stochastically simulated whether initial attack was successful (i.e., fully contained) using a Bernoulli simulation with the calculated probability described above. For the Progressive suppression scenario, we assumed ignitions were managed without any initial attack but with subsequent suppression.

For fires in which initial attack was not successful, we simulated subsequent suppression, which generally proceeds simultaneously from all sides of the fire (7, 22) and decreases in effectiveness as fireline intensity increases (23). We used suppression functions which use fireline intensity (I_θ) to determine the proportion of fire spread that would be suppressed at a given angle θ from the ignition (S_θ) (Fig. S12):

$$S_{maximum_\theta} = \exp\left(\frac{-I_\theta}{1500}\right)$$

$$S_{high_\theta} = 0.75 \cdot \exp\left(\frac{-I_\theta}{800}\right)$$

$$S_{moderate_\theta} = 0.5 \cdot \exp\left(\frac{-I_\theta}{300}\right)$$

$$S_{progressive_\theta} = \left(1 - \exp\left(\frac{-I_\theta}{150}\right)\right) \cdot \exp\left(\frac{-I_\theta}{1500}\right)$$

Maximum suppression roughly equates to the maximum possible effectiveness of on-the-ground fire suppression efforts, where suppression becomes virtually impossible past a certain fireline intensity (23).

Final daily distance burned under suppression (D_θ) was calculated as the unsuppressed distance burned ($D_\theta{}'$) multiplied by the proportion of fire remaining after suppression:

$$D_{maximum_\theta} = D_\theta{}' \cdot \left(1 - S_{maximum_\theta}\right)$$

$$D_{high_\theta} = D_\theta{}' \cdot (1 - S_{high_\theta})$$

$$D_{moderate_\theta} = D_\theta{}' \cdot (1 - S_{moderate_\theta})$$

$$D_{progressive_\theta} = D_\theta{}' \cdot (1 - S_{progressive_\theta})$$

Points on the ellipse were considered permanently extinguished if the daily distance burned was less than 5 m. Other points on the ellipse could continue burning, and a fire was not considered extinguished until points at all angles were extinguished or until after the 150th day of the fire season.

Simulation structure

We ran simulations where we varied either mean fuel aridity or fuel loading, while holding the other at constant mean values. A single simulation replicate involved 1000 ignitions, each of which had a unique, randomly simulated ignition day and timeseries of fuel moisture values and windspeeds (i.e., weather scenario). Using this ignition-day and weather scenario, we then simulated fire spread independently for either 1) all levels of fuel loading (n = 25; Table S1), while holding mean fire season fuel aridity constant (VPD of 1.17 kPa), or 2) all levels of mean fuel aridity (n = 25), while holding fuel loading constant (100-hr fuel loading of 11.23 Mg ha^{-1}). For each of these simulated fire-spread events, we also modeled all four scenarios of fire suppression (Maximum, High, Moderate, and Progressive) in addition to the no-suppression scenario. We then replicated a single simulation 40 times, for a total of 5 million simulated fires across each range of mean fuel aridity or fuel loading (1000 ignitions × 25 levels of fuel aridity or fuel loading × 5 levels of suppression × 40 replicates) (Fig. S13).

Processing simulations

To assess the effect of fire suppression on patterns of fire severity, we calculated the mean fire severity of fires, weighted by area (e.g., if two ha burned at CBI values of 2.0, and one ha burned at a CBI value of 1.0, the mean fire severity is 1.667). We also calculated the proportion of each fire that burned at high severity, defining "high-severity" fire as CBI values ≥ 2.25 (*sensu* 17). Finally, we calculated the required change in fuel aridity or fuel load for an unsuppressed fire to have the same mean fire severity (i.e., how much would conditions have to be different for an unsuppressed fire to yield the same mean fire severity?). We divided this absolute value by estimated yearly rates of change in mean fuel aridity and fuel loading, to evaluate how many years it would take for this change to be realized by climate change or fuel accumulation, respectively. To estimate a rate of VPD change, we used Ficklin & Novick's (2) projected

median increase in summer VPD of 0.72 kPa for the continental U.S. from the historical period (1979–2013; midpoint 1997) to the future period (2065–2099; midpoint 2082). We calculated the mean annual increase between midpoint years—0.00837 kPa $year^{-1}$—and used this to convert changes in mean fire season VPD to years. To calculate an average increase in 100-hr fuel accumulation, we estimated the slope of modeled dead wood carbon accumulation in the Western U.S. from 1980–2010 from Boisramé and colleagues (26), and assumed that carbon made up 50% of total fuel weight and 100-hr fuels comprised 50% of all dead fuels (in keeping with ratios of Fuel Model 10 (8)). This yielded a yearly 100-hr fuel load increase of 0.036 Mg ha^{-1} yr^{-1}, which we used to convert changes in 100-hr fuel load to years.

To investigate how fire suppression impacted patterns of burned area, we calculated the total area burned for each fire. We also calculated the average multiplicative yearly rate of increase (Δ) in burned area across the 240-year ranges of fuel aridity and fuel loading, where A_{max} is the area burned under maximum fuel aridity or fuel loading, respectively, A_{min} is the area burned under the minimum fuel aridity or fuel loading, respectively.

$$\Delta = \left(\frac{A_{max}}{A_{min}}\right)^{\frac{1}{240}}$$

We calculated the diversity of fire effects for each fire using the method detailed by Steel and colleagues (27), with CBI as the single input fire trait—which is equivalent to the mean absolute deviation of CBI values. We investigated how equally burned area was spread across burning days by calculating Lorenz curves (28) of daily area burned under each suppression scenario.

Within a replicate (i.e., 1000 ignitions × 25 fuel aridity or fuel loading levels; Fig. S14), for each suppression scenario we calculated the mean values for the parameters described above, at each fuel aridity or fuel loading level. We then calculated overall means across the 40 simulation replications and 95% confidence intervals to describe variability. We conducted all simulation and analysis in R (29).

Supplementary Figures

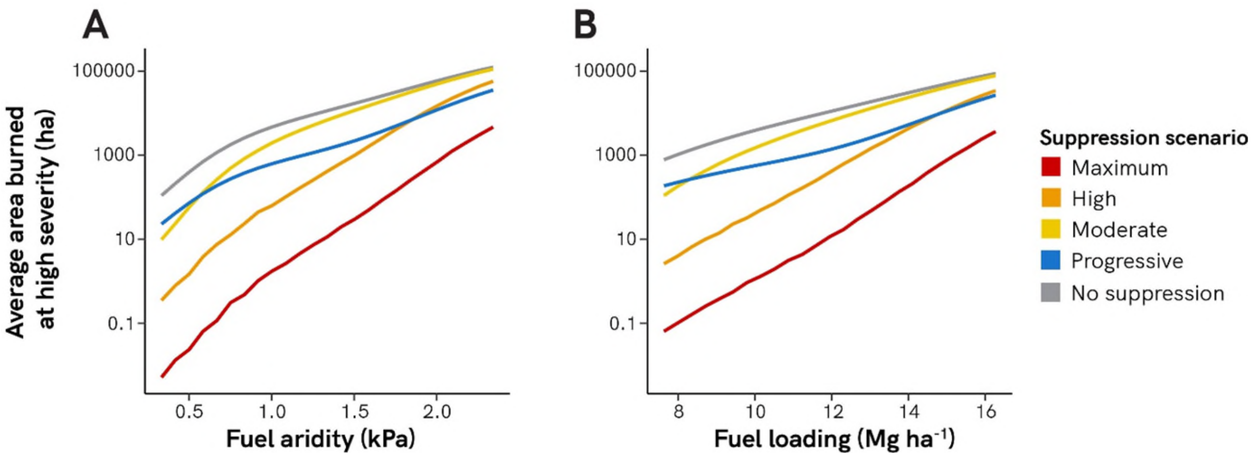

Figure S1. Average area burned at high severity across ranges of fuel aridity and fuel loading. Fuel loading in panel B depicts 100-hr surface fuel loading values. Variability across the 40 simulation replications is shown with 95% confidence intervals (too small to see on some curves). Simulations across the fuel aridity range were run at a constant 100-hr surface fuel loading of 11.23 Mg ha^{-1}; simulations across the fuel loading range were run at constant mean fire season fuel aridity of 1.17 kPa VPD.

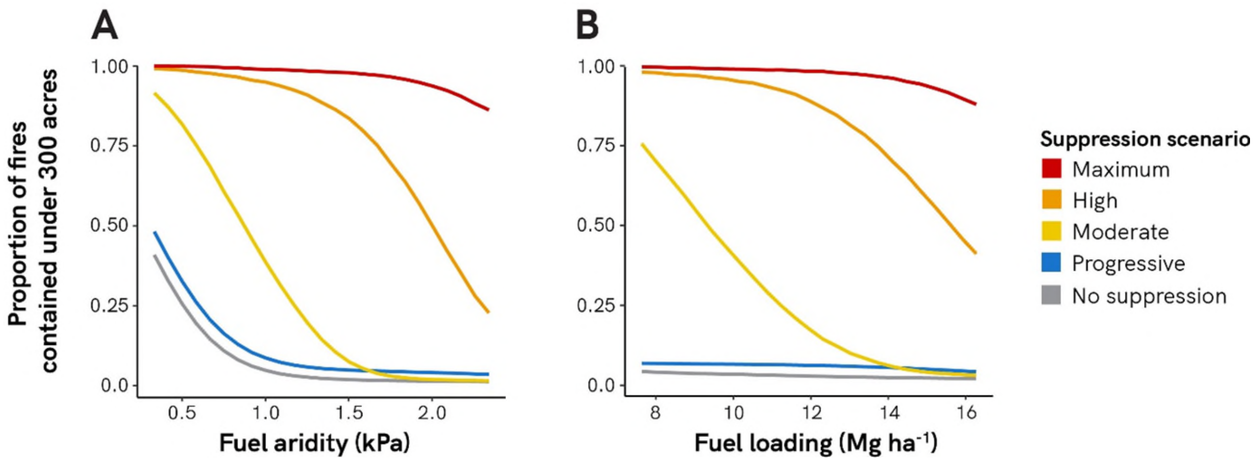

Figure S2. Average proportion of fires contained under 121 ha (300 acres) across ranges of fuel aridity and fuel loading. Fuel loading in panel B depicts 100-hr surface fuel loading values. Variability across the 40 simulation replications is shown with 95% confidence intervals (too small to see on some curves). Simulations across the fuel aridity range were run at a constant 100-hr surface fuel loading of 11.23 Mg ha^{-1}; simulations across the fuel loading range were run at constant mean fire season fuel aridity of 1.17 kPa VPD.

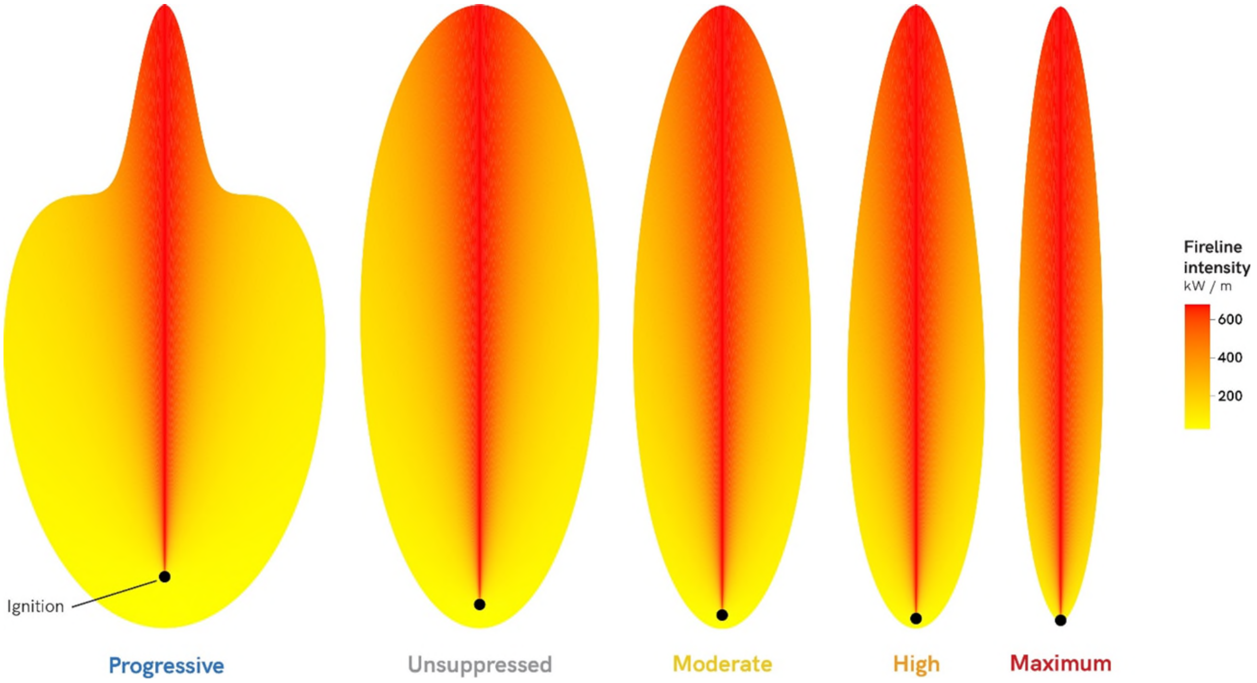

Figure S3. Though suppression decreases fire size, when expanded to equivalent lengths, fires managed with regressive suppression have a larger proportion of total area that comes from higher intensity fire. Example fires are identical to those presented in Fig. 1C in the main text. Simulations run at the following values: surface fuels using Fuel Model 10 (8); fuel moisture (litter = 2%, 1-hr dead fuels = 2%, 10-hr dead fuels = 3%, 100-hr dead fuels = 4%, live herbaceous = 70%, live woody = 100%); crown fuel (canopy bulk density = 0.15, fuel moisture content = 100%, canopy base height = 3 m, canopy fuel loading = 1 kg m^{-2}); slope = 0°; windspeed = 30 km hr^{-1}; wind adjustment factor = 0.15.

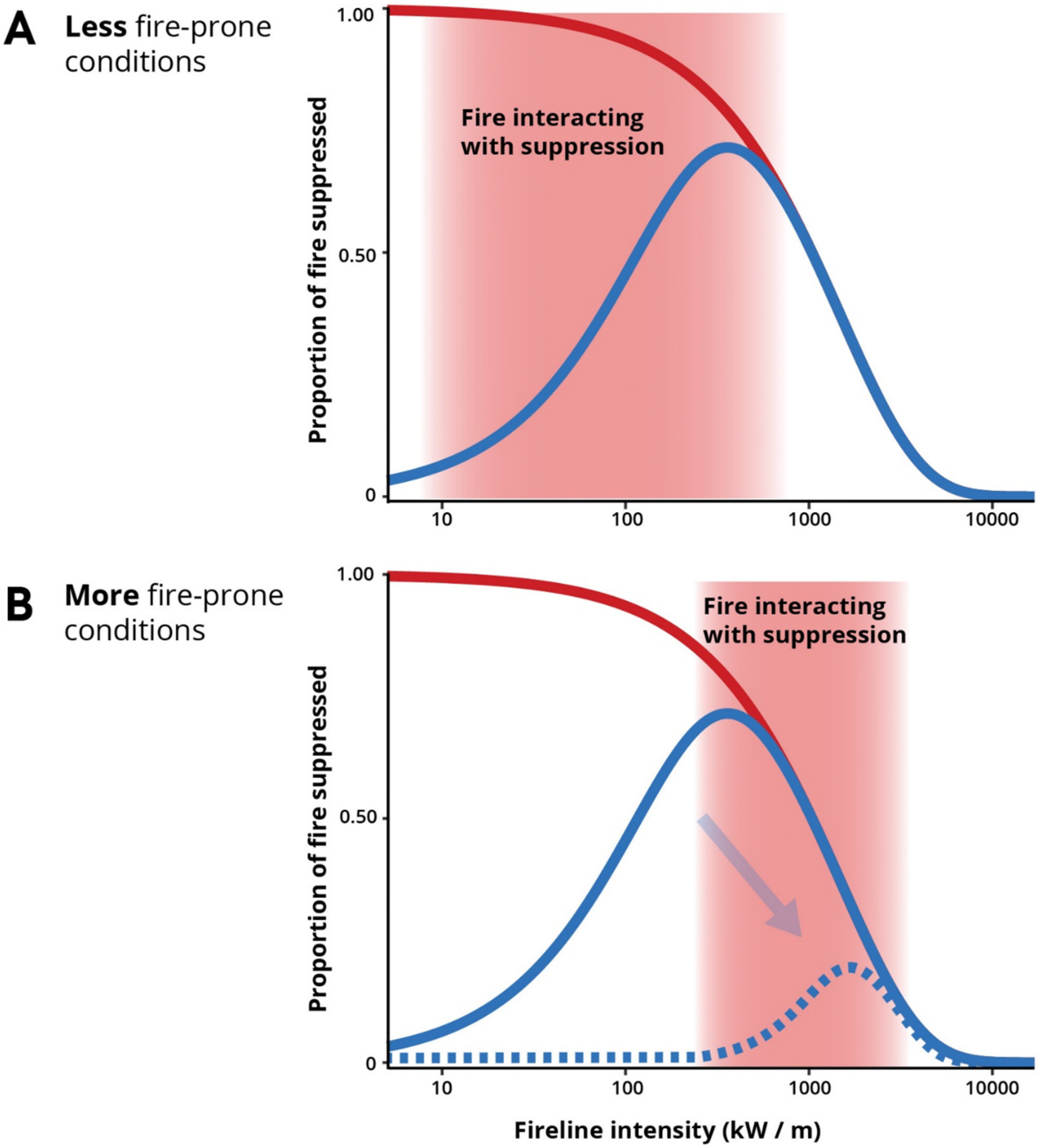

Figure S4. Conceptual figure of progressive suppression becoming less effective under extreme fire conditions. A) Under less fire-prone conditions, the Progressive suppression scenario (blue) removes more high-intensity fire than it does low-intensity fire, thus creating a moderating suppression bias. B) However, under more extreme fire conditions, the fires that interact with suppression are much higher intensity, and the Progressive suppression scenario now removes more low-intensity fire than it does high-intensity fire, becoming essential identical to Maximum regressive suppression (red). It is possible for a new suppression scenario (dashed blue line) that still maintains progressive suppression, but the operating space is continually reduced as conditions become more extreme.

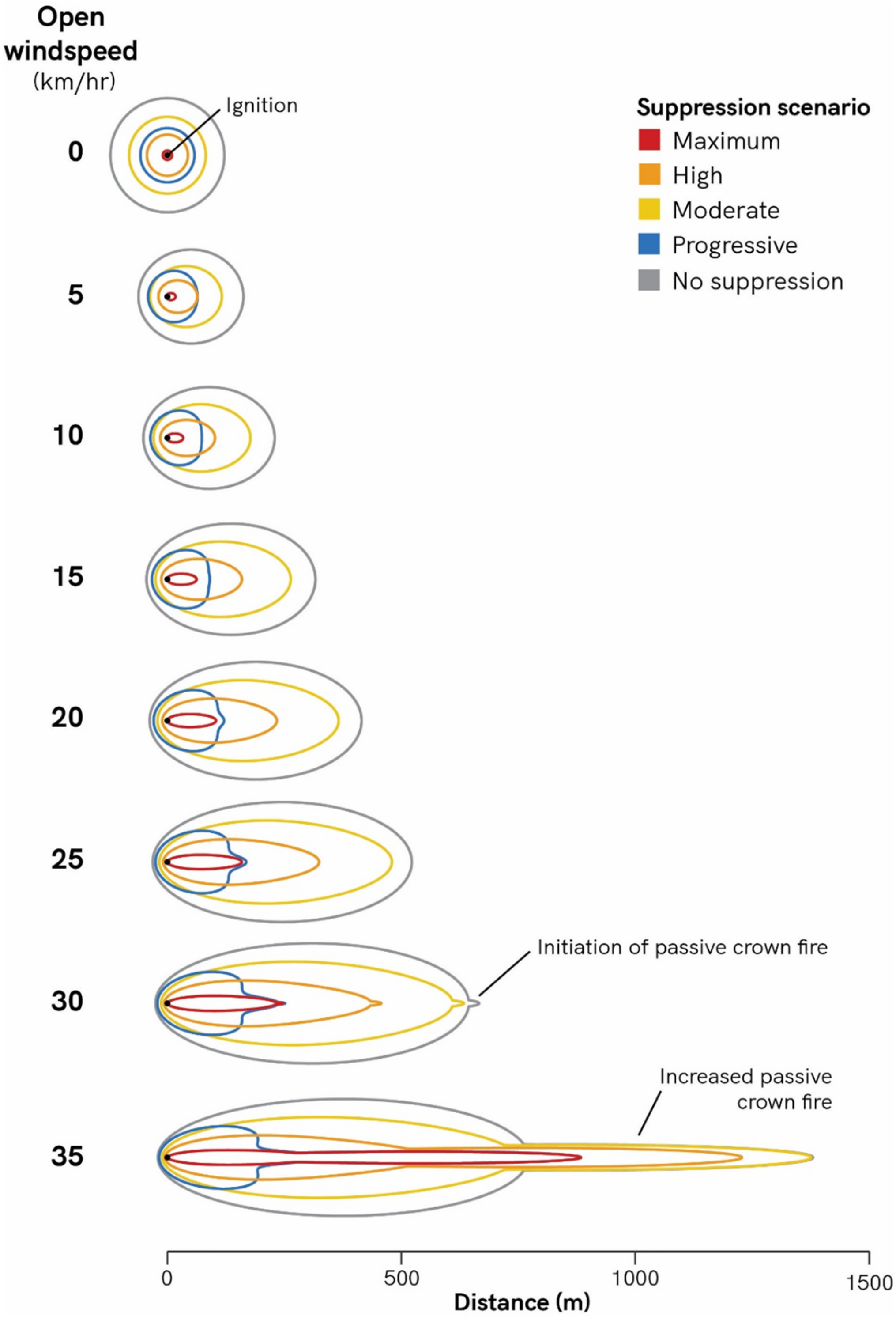

Figure S5. Fire perimeters after one day of burning with varying windspeed. Simulations run at the following values: surface fuels using Fuel Model 10 (8); fuel moisture (litter = 2%, 1-hr dead fuels = 2%, 10-hr dead fuels = 3%, 100-hr dead fuels = 4%, live herbaceous = 70%, live woody = 100%); crown fuel (canopy bulk density = 0.15, fuel moisture content = 100%, canopy base height = 2.5 m, canopy fuel loading = 1 kg m^{-2}); slope = 0°; wind adjustment factor = 0.15.

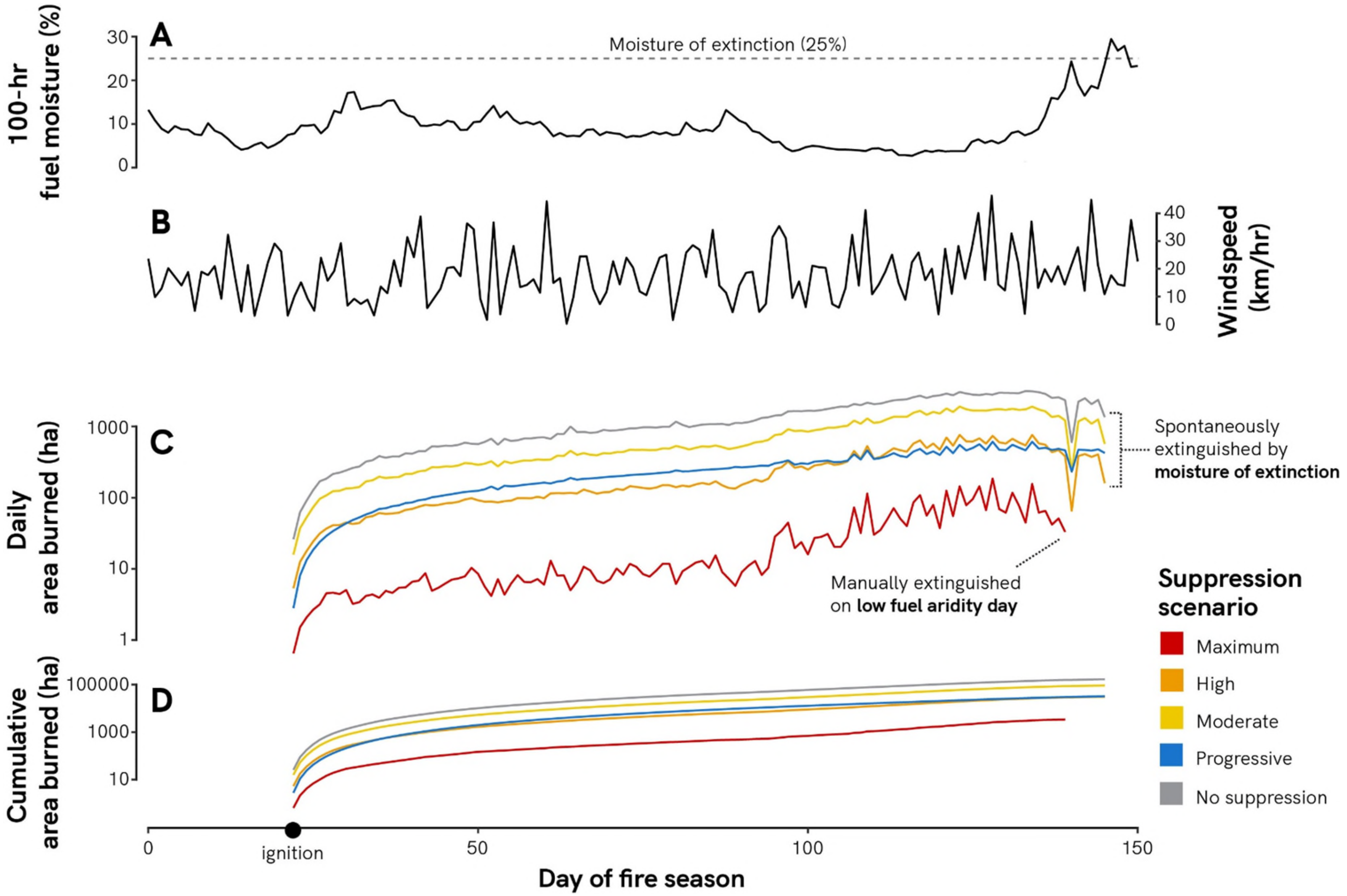

Figure S6. Simulated fire weather and spread for an example ignition across the 150-day fire season. A) Daily 100-hr surface fuel moisture. Fires do not spread on days above the moisture of extinction (25% fuel moisture); B) Windspeed; C) Daily area burned for fires managed under differing suppression scenarios (Maximum, High, Moderate, Progressive, and No suppression). Fires can be manually extinguished by suppression (i.e., all points on the ellipse spread less than 5 m on a given day) or they are automatically extinguished if the 100-hr fuel moisture rises above 25%. D) Cumulative area burned.

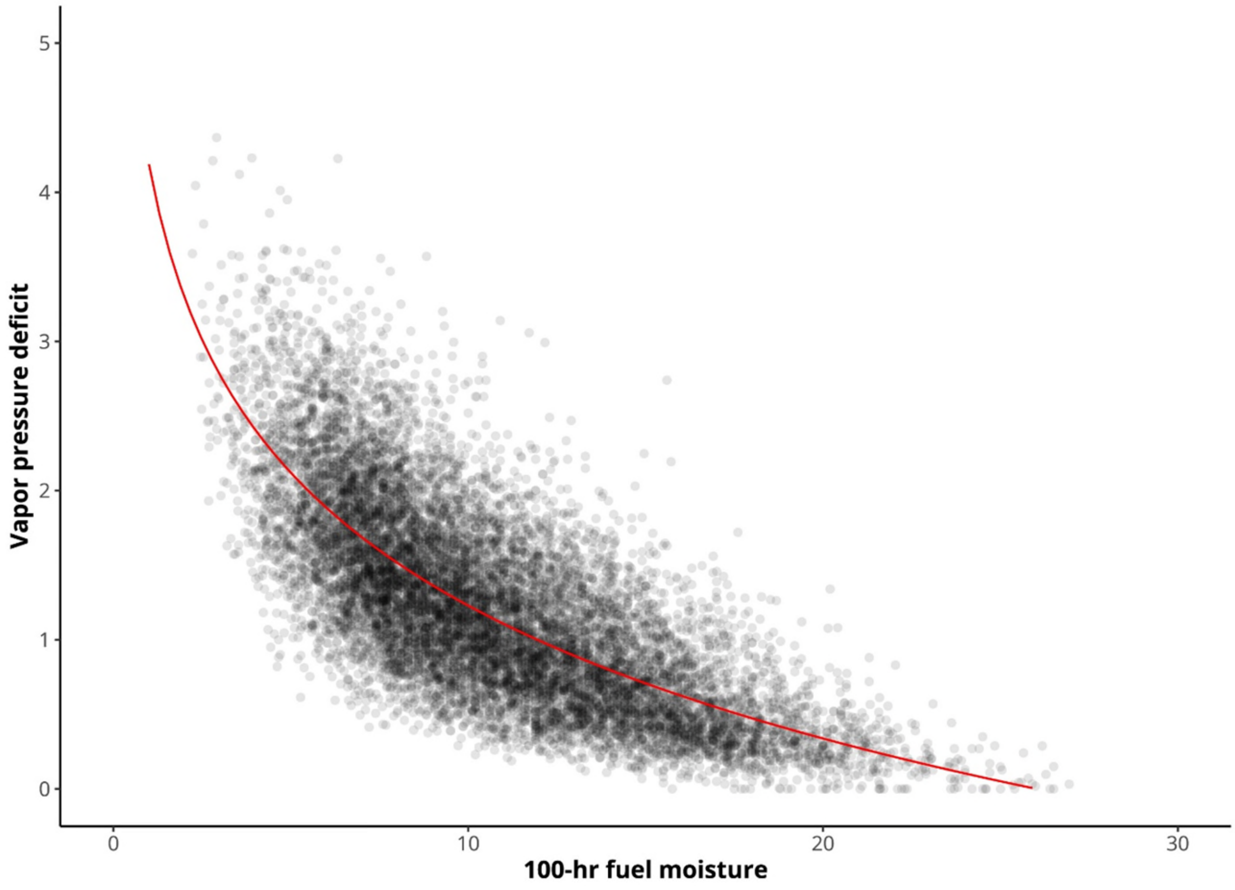

Figure S7. Exponential model fit (red line) of daily vapor pressure deficit as a function of daily 100-hr fuel moisture. Points shown for a random subset of 12,500 daily values. The model equation is $VPD = 4.188 - 1.285\, log(FM100)$; with $R^2 = 0.56$.

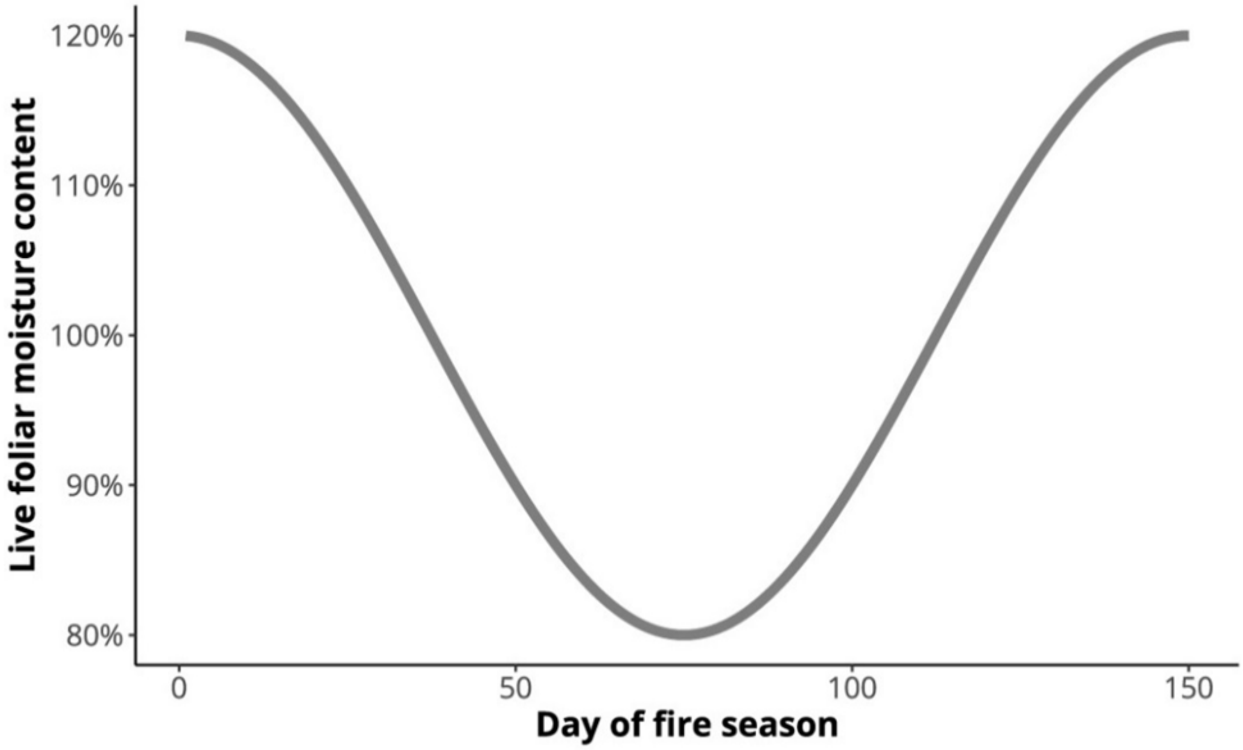

Figure S8. Simulated live foliar moisture content across the fire season.

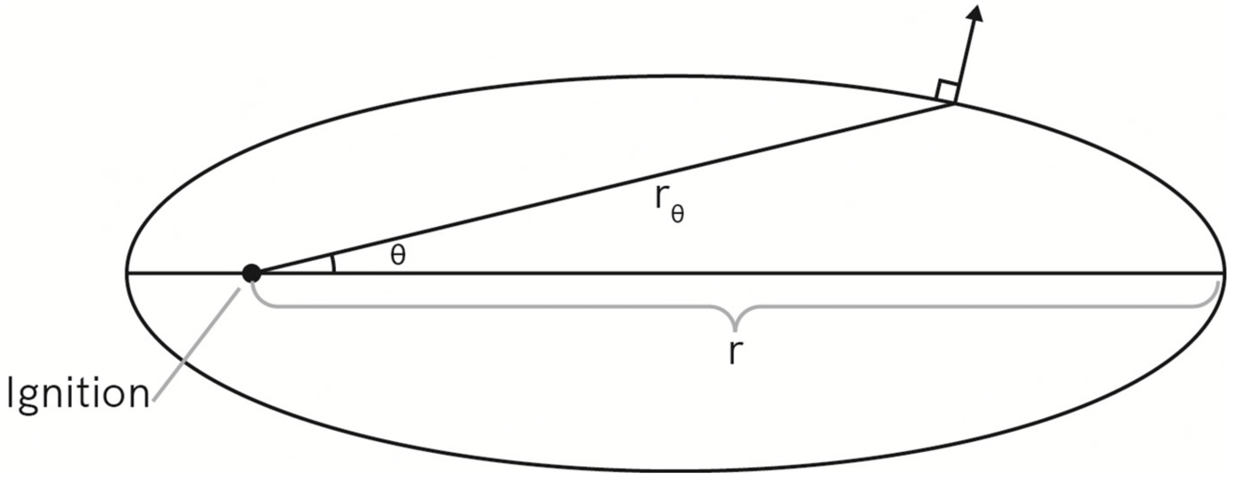

Figure S9. Elliptical shape parameters. The ignition is assumed to be at the rear focus of the ellipse. Fireline intensity (I_θ) and flame length (L_θ) were calculated in the direction of spread, normal to the fire perimeter, as indicated by the arrow.

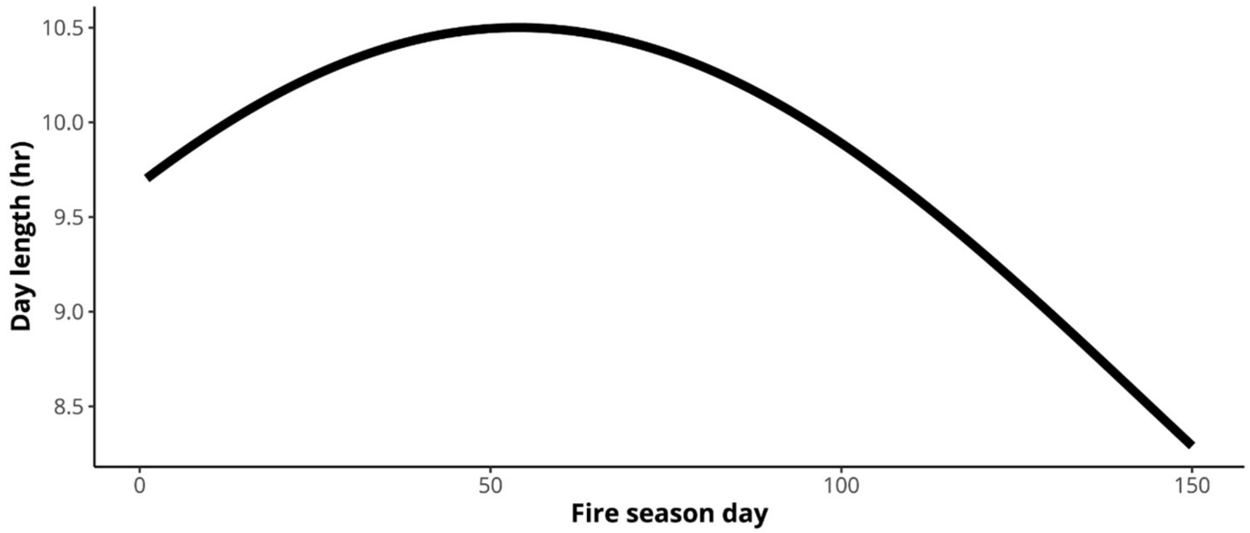

Figure S11. Modeled day length across the fire season.

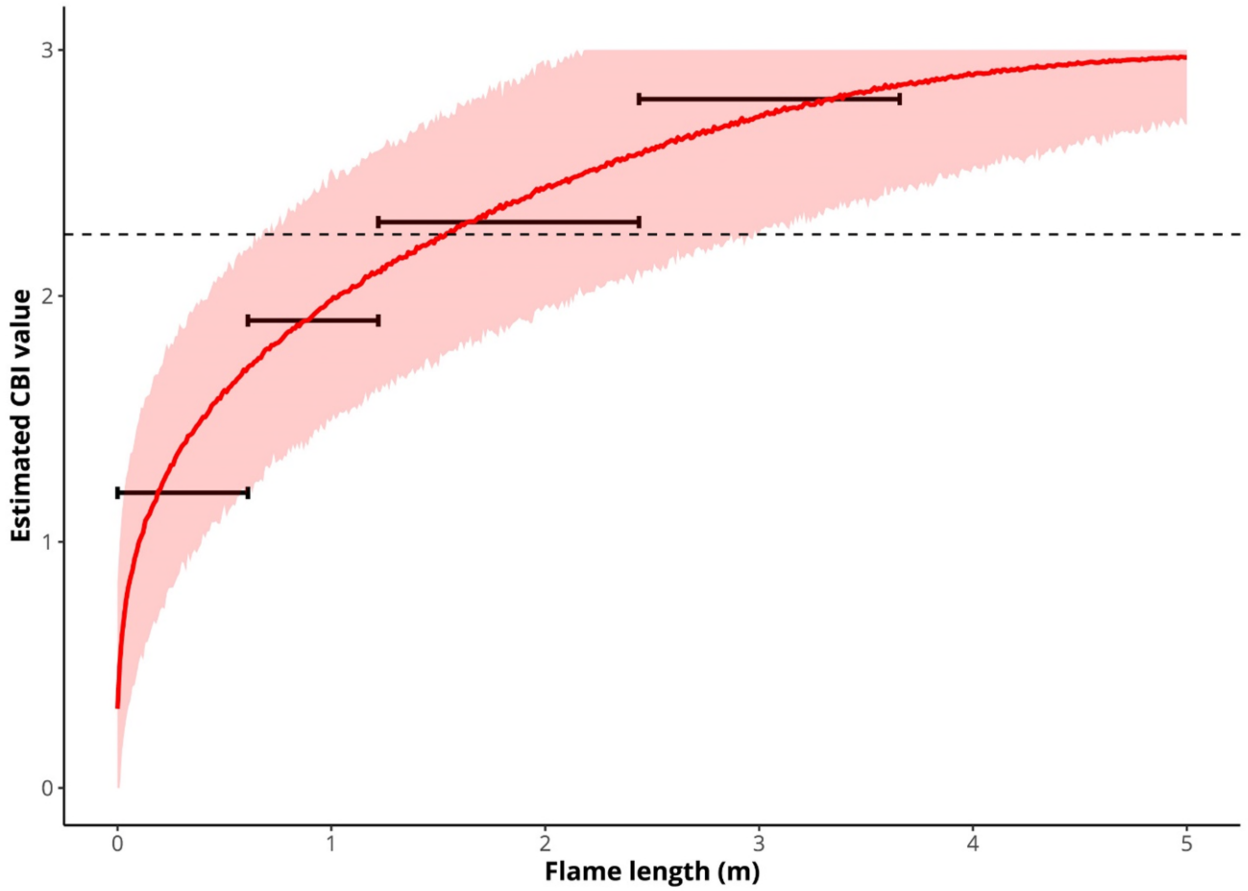

Figure S12. Simulated relationship between flame length and the Composite Burn Index (CBI). The mean value is denoted by the red line, while 95% of values fall within the red shaded region. Estimated CBI values for flame length classes are shown by the grey intervals. The CBI threshold for high-severity of 2.25 is shown with a dashed line.

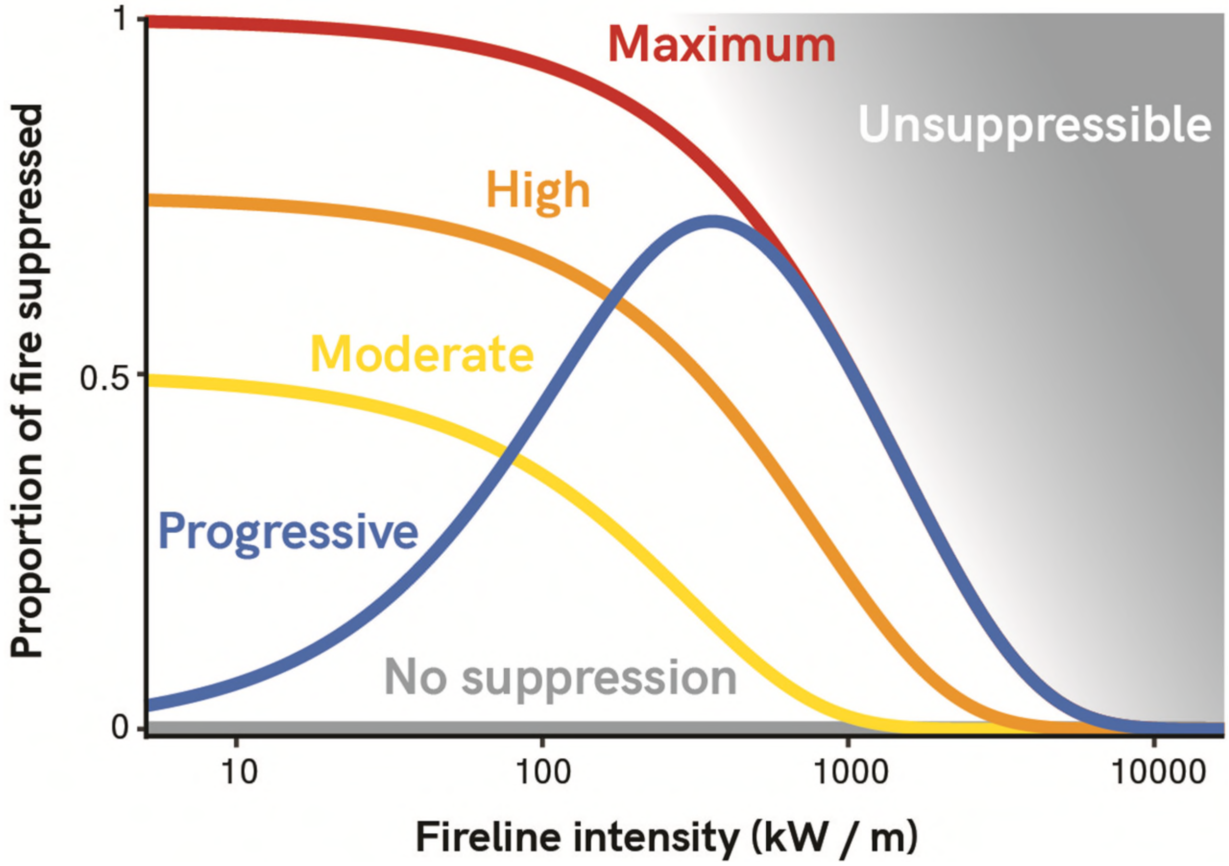

Figure S13. Proportion of fire suppressed for fires that escape initial attack. Colors depict the suppression scenarios used in the simulation. Beyond the Maximum suppression curve, fire intensity becomes too high to suppress fires.

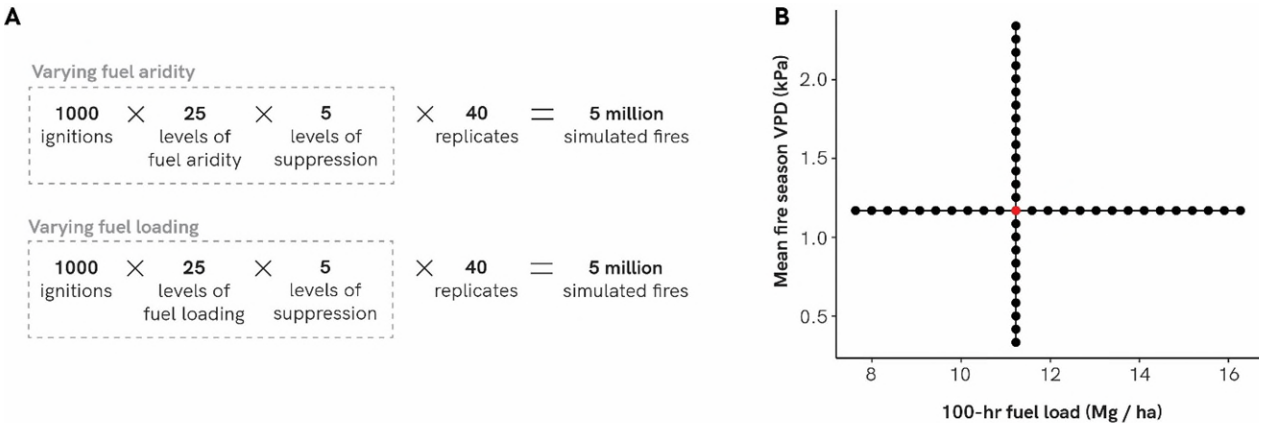

Figure S14. Simulation structure. A) Number of simulated fires for simulations across ranges of fuel aridity and fuel loading. B) Mean fire season VPD and 100-hr fuel load parameters of simulations. Note that *x* and *y* axes display one indicator input as a proxy for fuel aridity and fuel loading, respectively. Other model inputs of fuel aridity (e.g., 1-hr, 10-hr fuel moisture) and fuel loading (e.g., 1-hr, 10-hr dead surface fuel loads) were calculated as described above.

Supplementary Tables

Table S1: Varying surface and canopy fuel parameters of the varying 25 fuel sets. All other parameters were held constant (surface fuels: litter = 0 Mg ha^{-1}, live herbaceous fuel = 0 Mg ha^{-1}, surface-area-to-volume ratio (SAV) 1-hour = 6562, SAV 10-hour = 358, SAV 100-hour = 98, SAV live woody = 4921, moisture of extinction of dead fuels = 25%, heat content = 18622 kJ kg^{-1}; canopy fuels: canopy base height = 2.0 m). The surface fuels of fuel set 11 (highlighted cells) correspond to the values of Fuel Model 10 (8).

	Surface fuels					*Canopy fuels*	
**Fuel set**	**1-hr fuel** Mg ha^{-1}	**10-hr fuel** Mg ha^{-1}	**100-hr fuel** Mg ha^{-1}	**Live woody fuel** Mg ha^{-1}	**Fuel bed depth** cm	**Canopy fuel loading** kg m^{-2}	**Canopy bulk density** kg m^{-3}
1	4.57	3.04	7.63	3.04	20.71	0.060	0.5
2	4.79	3.19	7.99	3.19	21.69	0.064	0.6
3	5.00	3.33	8.35	3.33	22.66	0.069	0.7
4	5.22	3.47	8.71	3.47	23.64	0.073	0.8
5	5.44	3.62	9.07	3.62	24.62	0.077	0.9
6	5.65	3.76	9.43	3.76	25.59	0.082	1.0
7	5.87	3.91	9.79	3.91	26.57	0.086	1.1
8	6.08	4.05	10.15	4.05	27.55	0.090	1.2
9	6.30	4.19	10.51	4.19	28.53	0.095	1.3
10	6.51	4.34	10.87	4.34	29.50	0.099	1.4
11	6.73	4.48	11.23	4.48	30.48	0.103	1.5
12	6.95	4.62	11.59	4.62	31.46	0.108	1.6
13	7.16	4.77	11.95	4.77	32.43	0.112	1.7
14	7.38	4.91	12.31	4.91	33.41	0.116	1.8
15	7.59	5.05	12.67	5.05	34.39	0.121	1.9
16	7.81	5.20	13.03	5.20	35.37	0.125	2.0
17	8.02	5.34	13.39	5.34	36.34	0.129	2.1
18	8.24	5.49	13.75	5.49	37.32	0.134	2.2
19	8.46	5.63	14.11	5.63	38.30	0.138	2.3
20	8.67	5.77	14.47	5.77	39.27	0.142	2.4
21	8.89	5.92	14.83	5.92	40.25	0.147	2.5
22	9.10	6.06	15.19	6.06	41.23	0.151	2.6
23	9.32	6.20	15.55	6.20	42.21	0.155	2.7
24	9.53	6.35	15.91	6.35	43.18	0.160	2.8
25	9.75	6.49	16.27	6.49	44.16	0.164	2.9

Table S2: Estimates of burn severity values for each CBI strata as a function of flame length classes. Ryan and Noste (1985) provide flame length classes in feet; we thus provide flame lengths in feet as well as in meters. Final CBI values are averages of all five strata values.

		CBI strata severity					
**Flame length (ft)**	**Flame length (m)**	**A. Substrates**	**B. Seedlings**	**C. Saplings**	**D. Pole-size trees**	**E. Big trees (small and large saw timber)**	**CBI**
0–2	0–0.6	1.0	2.0	1.5	1.0	0.5	**1.2**
2–4	0.6–1.2	1.5	3.0	2.5	1.5	1.0	**1.9**
4–8	1.2–2.4	2.0	3.0	3.0	2.0	1.5	**2.3**
8–12	2.4–3.7	2.5	3.0	3.0	3.0	2.5	**2.8**
>12	>3.7	3.0	3.0	3.0	3.0	3.0	**3.0**

Supplementary References

1. M. A. Finney, C. W. McHugh, I. C. Grenfell, K. L. Riley, K. C. Short, A simulation of probabilistic wildfire risk components for the continental United States. *Stoch Environ Res Risk Assess* **25**, 973–1000 (2011).

2. D. L. Ficklin, K. A. Novick, Historic and projected changes in vapor pressure deficit suggest a continental-scale drying of the United States atmosphere. *Journal of Geophysical Research: Atmospheres* **122**, 2061–2079 (2017).

3. NWCG, “Fire Behavior Field Reference Guide” (National Wildfire Coordinating Group, 2021) (February 14, 2023).

4. The Nature Conservancy, Terrestrial Ecoregions (2019) (March 7, 2023).

5. N. Gorelick, *et al.*, Google Earth Engine: Planetary-scale geospatial analysis for everyone. *Remote Sensing of Environment* **202**, 18–27 (2017).

6. J. T. Abatzoglou, Development of gridded surface meteorological data for ecological applications and modelling. *International Journal of Climatology* **33**, 121–131 (2011).

7. M. A. Finney, S. S. McAllister, T. P. Grumstrup, J. M. Forthofer, *Wildland Fire Behaviour* (CSIRO Publishing, 2021).

8. H. E. Anderson, Aids to determining fuel models for estimating fire behavior. *Gen. Tech. Rep. INT-122. Ogden, Utah: U.S.Department of Agriculture, Forest Service, Intermountain Forest and Range Experiment Station. 22p.* **122** (1982).

9. R. C. Rothermel, A mathematical model for predicting fire spread in wildland fuels. *Res. Pap. INT-115. Ogden, UT: U.S. Department of Agriculture, Intermountain Forest and Range Experiment Station. 40 p.* **115** (1972).

10. F. A. Albini, Estimating wildfire behavior and effects. *Gen. Tech. Rep. INT-GTR-30. Ogden, UT: U.S. Department of Agriculture, Forest Service, Intermountain Forest and Range Experiment Station. 92 p.* **30** (1976).

11. M. A. Finney, FARSITE: Fire Area Simulator-model development and evaluation. *Res. Pap. RMRS-RP-4, Revised 2004. Ogden, UT: U.S. Department of Agriculture, Forest Service, Rocky Mountain Research Station. 47 p.* **4** (1998).

12. J. P. Ziegler, C. M. Hoffman, W. Mell, firebehavioR: An R Package for Fire Behavior and Danger Analysis. *Fire* **2**, 41 (2019).

13. D. H. Anderson, E. A. Catchpole, N. J. D. Mestre, T. Parkes, Modelling the spread of grass fires. *The ANZIAM Journal* **23**, 451–466 (1982).

14. A. J. Simard, A. Young, R. Redmond, “Airpro: an air tanker productivity computer simulation model application” (Canadian Forestry Service, Forest Fire Research Institute, 1978) (August 13, 2022).

15. P. L. Andrews, The Rothermel surface fire spread model and associated developments: A comprehensive explanation. *Gen. Tech. Rep. RMRS-GTR-371. Fort Collins, CO: U.S. Department of Agriculture, Forest Service, Rocky Mountain Research Station. 121 p.* **371** (2018).

16. J. Scott, E. Reinhardt, Assessing Crown Fire Potential by Linking Models of Surface and Crown Fire Behavior. *USDA Forest Service - Research Paper RMRS-RP* (2001).

17. E. A. Catchpole, N. D. Mestre, A. Gill, Intensity of fire at its perimeter. *Australian forest research* (1982) (October 3, 2023).

18. C. H. Key, N. C. Benson, Landscape Assessment: Sampling and Analysis Methods. *USDA Forest Service General Technical Report* **RMRS-GTR-164-CD**, 55 (2006).

19. K. Ryan, N. Noste, Evaluating Prescribed Fires. *Proceedings - Symposium and Workshop on Wilderness Fire*, 230–238 (1985).

20. S. A. Parks, J. T. Abatzoglou, Warmer and Drier Fire Seasons Contribute to Increases in Area Burned at High Severity in Western US Forests From 1985 to 2017. *Geophysical Research Letters* **47**, e2020GL089858 (2020).

21. M. P. Plucinski, Fighting Flames and Forging Firelines: Wildfire Suppression Effectiveness at the Fire Edge. *Curr Forestry Rep* **5**, 1–19 (2019).

22. M. P. Plucinski, Contain and Control: Wildfire Suppression Effectiveness at Incidents and Across Landscapes. *Curr Forestry Rep* **5**, 20–40 (2019).

23. P. Andrews, F. Heinsch, L. Schelvan, How to Generate and Interpret Fire Characteristics Charts for Surface and Crown Fire Behavior. *USDA Forest Service - General Technical Report RMRS-GTR* (2011).

24. M. C. Arienti, S. G. Cumming, S. Boutin, Empirical models of forest fire initial attack success probabilities: the effects of fuels, anthropogenic linear features, fire weather, and management. *Can. J. For. Res.* **36**, 3155–3166 (2006).

25. J. S. Fried, *et al.*, Predicting the effect of climate change on wildfire behavior and initial attack success. *Climatic Change* **87**, 251–264 (2008).

26. G. F. S. Boisramé, T. J. Brown, D. M. Bachelet, Trends in western USA fire fuels using historical data and modeling. *Fire Ecology* **18**, 8 (2022).

27. Z. L. Steel, B. M. Collins, D. B. Sapsis, S. L. Stephens, Quantifying pyrodiversity and its drivers. *Proceedings of the Royal Society B: Biological Sciences* **288**, 20203202 (2021).

28. M. O. Lorenz, Methods of Measuring the Concentration of Wealth. *Publications of the American Statistical Association* **9**, 209–219 (1905).

29. R Core Team, R: A language and environment for statistical computing. (2023).
